# Communication

**Published:** 1876-06

**Authors:** J. B. Williams

**Affiliations:** Charleston, N. C.


We have received the following letter from an eminent Southern physician, which contains some practical suggestions of interest to many:

CHARLESTON, N. C., April 24th, 1876.

To The Editor of Hall's Journal of Health:

*Dear Sir:* Your valuable Journal is greatly prized by its many readers in this section of the country. Your article in the March number, page 137, pleased me very much. I have been thinking of writing, urging you to say *more*, and *all you can*, in behalf of *suffering, dying infants.* Thousands of children, and particularly infants, die every year from *vitiated* air, the *bad breaths* of their parents, by day and by night. The mother, while suckling the child, breathes—yes, blows—her sickening breath into the child's face until it frets, puts up its little hands, finally, unable to endure the *offence*, lets go of the breast and turns away, gasping for pure air. How often do we see this? and yet the fond, ignorant mother, utterly unconscious of what she is doing, the more it struggles and cries, the closer she *holds* it to her breast, until it sickens and dies, and that, too, from intended kindness; and, I am sorry to add, its death is often hastened by injurious medical treatment. Will you please hand the $1, which I indorse in this letter, to Hall & Ruckel, and request them to send me at once some more of the "Van Deren Remedy" for summer complaint of children? I used it last summer with the most satisfactory results, never having lost a little patient out of the thirty cases that I treated. It is *my remedy* for all bowel complaints of children.

Yours truly, J. B. Williams.

